# Acute Combination of Nitrogen Deprivation and High Irradiance Induces the Simultaneous Accumulation of Astaxanthin and Lutein in Continuous Cultures of the Microalga *Chromochloris zofingiensis*

**DOI:** 10.3390/plants15060902

**Published:** 2026-03-14

**Authors:** María Morales-Pineda, Mercedes García-González, M. Elena García-Gómez, Francisco J. Romero-Campero, Marcos Ramos-González

**Affiliations:** 1 Instituto de Bioquímica Vegetal y Fotosíntesis, Universidad de Sevilla-CSIC, Avda. Américo Vespucio, 49, 41092 Sevilla, Spain; maria.morales.pineda.sspa@juntadeandalucia.es (M.M.-P.); mggonza@us.es (M.G.-G.); maria.elena@ibvf.csic.es (M.E.G.-G.); 2 Department of Computer Science and Artificial Intelligence, Universidad de Sevilla, Av. Reina Mercedes s/n, 41012 Sevilla, Spain

**Keywords:** continuous culture, astaxanthin, lutein, nitrogen availability, *Chromochloris zofingiensis*

## Abstract

Carotenoids play a central role in photosynthesis and cellular protection, and microalgae represent a sustainable platform for their commercial production. Here, we optimized the accumulation of the high-value carotenoids astaxanthin and lutein in continuous photoautotrophic cultures of *Chromochloris zofingiensis* by modulating nitrogen supply and light intensity. Reducing nitrate availability strongly promoted astaxanthin accumulation, whereas lutein levels remained largely unaffected. For 4% N in the dry biomass, accumulation of astaxanthin was highest and that of lutein lowest, while the opposite was recorded for 9% N. Average irradiance positively affected lutein accumulation independently of nitrate, whereas that of astaxanthin only increased under nitrogen-limiting conditions. Integrated transcriptomics and carotenoid profiling analysis revealed nitrogen availability as the dominant regulatory factor, with a synergistic interaction with light that enhances their individual effects. Nitrate limitation redirected metabolic flux from lycopene toward β-carotene and its subsequent conversion to astaxanthin via BKT1 overexpression, while high irradiance induced CYP97A1 and CYP97C expression, favoring lutein biosynthesis. Together, these findings demonstrate that targeted control of nitrogen and light enables the continuous and programmable production of *C. zofingiensis* biomass with a specific astaxanthin-to-lutein ratio, highlighting its potential for industrial carotenoid bioprocesses.

## 1. Introduction

Microalgae possess properties characteristic of plants, such as efficient oxygenic photosynthesis and simple nutritional requirements, along with biotechnological attributes inherent to microbial cells, including rapid growth in liquid culture and the capacity to accumulate or secrete certain metabolites. Due to these unique properties, these microorganisms are well suited for a diversity of applied biotechnological processes [1].

Microalgae are a major natural source for a variety of high-value commercial compounds, including neutral lipid triacylglycerols, high-quality protein, polyunsaturated fatty acids, and a diversity of pigments for which these photosynthetic microorganisms represent an almost exclusive renewable resource [2,3,4].

Carotenoids have a role in light harvesting and are also able to photoprotect the photosynthetic machinery from excess light by scavenging reactive oxygen species [5]. Carotenoids exhibit a broad market prospect in aquaculture, nutraceuticals, foods and cosmetics due to their exceptional antioxidant activity, their relevance as precursors of vitamin A synthesis or as macular pigments and other physiological effects [6].

Lutein is a primary carotenoid, characterized by its importance in photosynthetic and light-harvesting processes and by its storage in plastids [7]. Astaxanthin, on the other hand, is a secondary carotenoid, which is derived from primary carotenoids and is related to signaling, growth, and development processes [7]. Both have been extensively explored for food, feed dyes and feed additives in the poultry and aquaculture industries and for nutraceutical and pharmaceutical uses [8,9,10]. Astaxanthin, due to having the highest antioxidant activity known in nature, has been related to several health benefits, including neuroprotective, cardioprotective and antitumoral properties. This suggests its therapeutic potential for the prevention or co-treatment of dementia, Alzheimer, Parkinson, cardiovascular diseases and cancer [11,12,13]. On the other hand, lutein is present in the retina of the eye, acting as a strong antioxidant and filtering high-energy blue light, suggesting its beneficial effects in ocular diseases such as age-related macular degeneration, diabetic retinopathy, retinopathy of prematurity, myopia and cataracts [14,15]. Furthermore, due to its antioxidant activity, it has potentially beneficial effects on fish larvae development [16].

The flowers of Marigold (*Tagetes erecta*) represent the major commercial source of lutein [17] but present biomass and seasonality limitations that have increased the interest in its production using microalgae. A wide range of these organisms have been observed to produce lutein, such as *Chlorella*, *Muriellopsis*, *Parachlorella* and *Acutodesmus* genus [18]. Species such as *Haematococcus pluvialis*, *Dunaliella salina* and *Chromochloris zofingiensis* are the most studied species for astaxanthin production. *H. pluvialis* is especially known for its capacity to produce astaxanthin, often in response to stress conditions such as nutrient limitation [19]. The molecular basis of this response has recently been revealed [20]. *D. salina* accumulates large amounts of β-carotene, which can be converted to astaxanthin [21].

*Chromochloris zofingiensis*, a freshwater green microalga, also referred as *Chlorella zofingiensis* or *Muriella zofingiensis*, is able to grow robustly to high cell densities under photoautotrophic, heterotrophic and mixotrophic conditions [22,23,24,25,26,27], with an interesting cellular content of both lutein and astaxanthin [21,28].

Thanks to the sequencing of the *C. zofingiensis* genome and the availability of genetic tools, the understanding of carotenogenic pathways in this microalga has been greatly improved [29]. Recently, transcriptomic studies on carotenogenesis have been carried out under different conditions, including glucose induction, nitrogen limitation or deprivation, and high light [27,29,30,31,32,33,34].

In general, culture conditions that promote astaxanthin accumulation exert the opposite effect on lutein. Considerable effort has been made towards finding a combination of culture factors that promote co-accumulation of both pigments in different organisms. However, this trade-off has been found to hinder simultaneous production of astaxanthin and lutein in many microalgae species, such as *Coelastrella* sp. [35], *H. pluvialis* [36] and *Muriellopsis* sp. [37]. In contrast, *C. zofingiensis* has been shown to be an exception to this rule, being able to accumulate both compounds under nutritional stress, to an extent [27]. Although limiting nutrients in the culture media seems to be a requisite to accumulate astaxanthin and reduces lutein accumulation, light stress has been successfully applied to the enhancement of lutein levels without compromising astaxanthin ones [38,39].

The present study was conducted to assess the potential of *C. zofingiensis* for the production of lutein, astaxanthin, or both carotenoids. The effect of changes in relevant culture parameters as dilution rate, nitrate concentration in culture media and available light intensity has been analyzed under a continuous growth regime in order to understand how these factors influence carotenoid biosynthesis and accumulation in this microalga. Additionally, the most appropriate conditions favoring the simultaneous accumulation of lutein and astaxanthin have been established as a relevant issue of applied interest. Moreover, the molecular basis of the regulation underlying the response to these factors has been studied by applying joint transcriptomics and carotenoid profiling analysis, unveiling differential responses for the individual carotenogenic enzymes. Using this approach, elements that serve as links between the individual stress responses have been identified, in which the combination of both factors gives rise to outcomes on gene expression or carotenoid accumulation that do not match the sum of their individual effects.

## 2. Results and Discussion

### 2.1. High Dilution Rate Favors Astaxanthin and Lutein Accumulation

**Figure 1:**
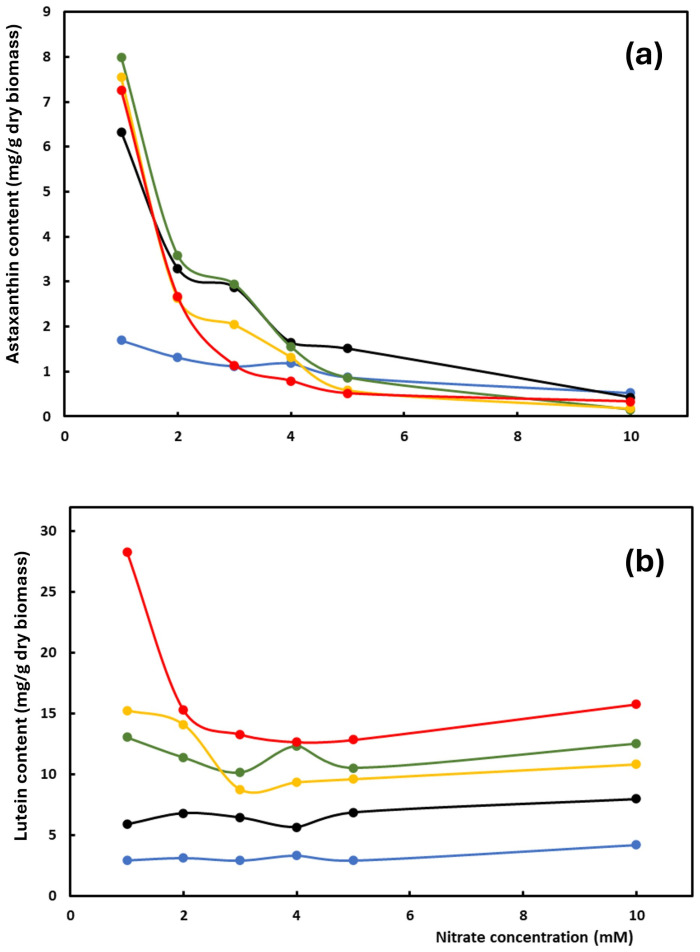
Influence of nitrate concentration in culture media on the content of (**a**) astaxanthin and (**b**) lutein in the biomass of continuous cultures of *C. zofingiensis* at different dilution rates: 0.1 d^−1^ (blue), 0.3 d^−1^ (black), 0.5 d^−1^ (green), 0.7 d^−1^ (yellow) and 0.9 d^−1^ (red).

The ability of *C. zofingiensis* to accumulate significant amounts of valuable carotenoids, namely lutein and astaxanthin, has been previously demonstrated under batch culture conditions [27]. In this study, the influence of dilution rate, nitrate supply, irradiance and specific nitrate input (SNI) on the content of lutein and astaxanthin in the biomass in the continuous culture of *C. zofingiensis* was analyzed. Astaxanthin content in the biomass gradually decreased when nitrate in the culture media increased from 1 to 10 mM for all tested dilution rates (Figure 1a). The higher astaxanthin content was reached at the higher dilution rates assayed (0.5 d^−1^, 0.7 d^−1^ and 0.9 d^−1^), with more than 7 mg astaxanthin/g dry biomass at 1 mM of nitrate in culture media. Cultures operating at a dilution rate greater than 0.9 d^−1^ were washed out. However, lutein content in the biomass remained constant when nitrate availability increased in the culture media, registering values greater than 10 mg lutein/g dry biomass at high dilution rates (0.5 d^−1^, 0.7 d^−1^, 0.9 d^−1^) (Figure 1b). Lutein accumulation rose as the dilution rate increased. It also significantly increased for the 10 mM condition in comparison with 5, 4 and 3 mM. In a batch culture, astaxanthin is preferably synthesized in the later stationary phase, when the nitrogen availability is limited [27], in a similar way to our conditions in a continuous culture with low concentrations of nitrate in the culture media.

The results reflect that both carotenoids are differentially accumulated, with lutein abundance being favored by non-limiting growth conditions (primary carotenoid) and that of astaxanthin (as a secondary carotenoid) by growth-restricted conditions.

### 2.2. Nitrogen Availability Greatly Impacts Carotenoid Content

**Figure 2:**
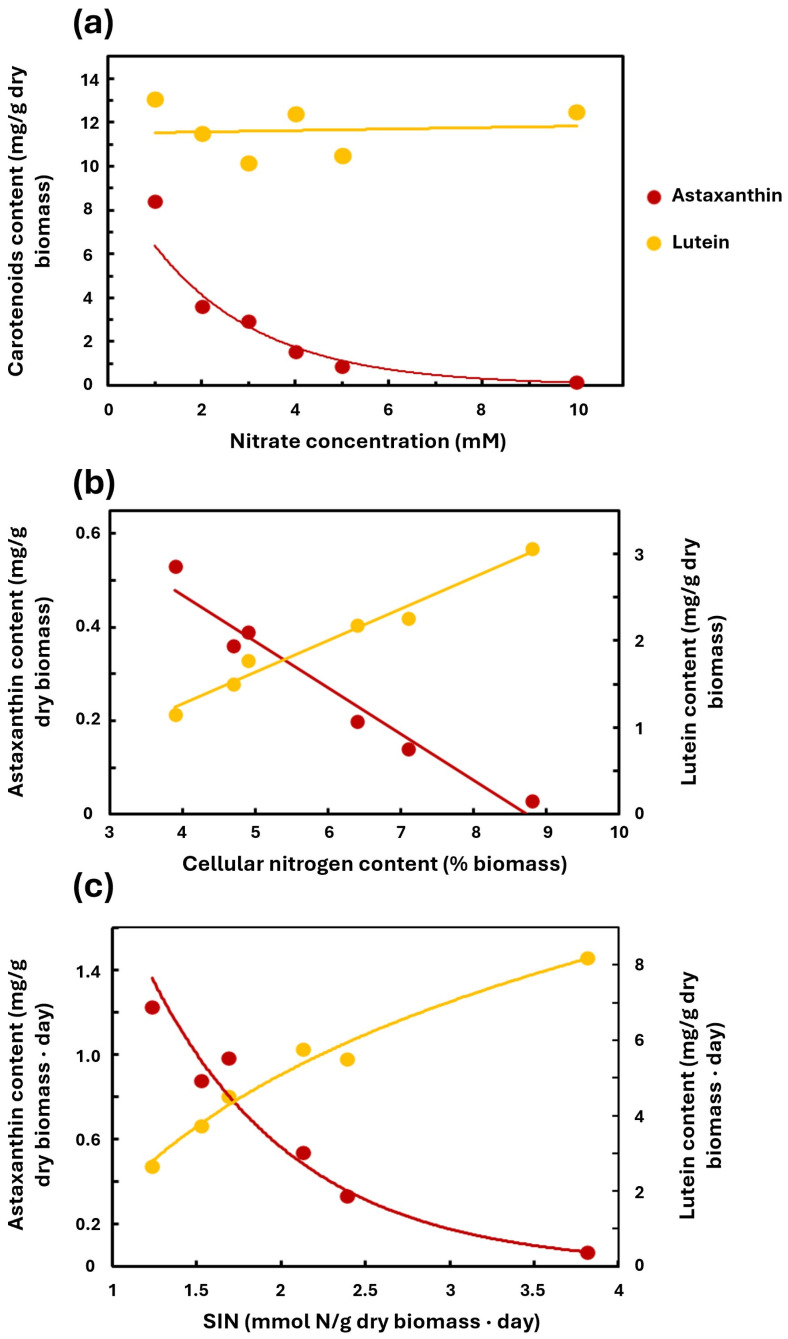
Effect of nitrate supply on the content of astaxanthin (red) and lutein (yellow). (**a**) Effect of nitrate concentration in the culture media on the content of each carotenoid per g of dry biomass. (**b**) Effect of cellular nitrogen content on the content of each carotenoid per g of dry biomass. (**c**) Effect of SNI on the content of each carotenoid per g of dry biomass by day. Measures taken in continuous culture at a dilution rate of 0.5 d^−1^. Culture conditions as in Figure 1. Content of astaxanthin is depicted in red and lutein in yellow.

For a specific dilution rate (0.5 d^−1^), the influence of the nitrate supply on the content of the different carotenoids in the dry biomass was analyzed. Lutein levels remained virtually unchanged (~11 mg/g dry weight) for this specific dilution rate despite significant variations in the nitrate concentration in the culture media (Figure 2a), albeit with slightly higher values for the 10 mM nitrate condition compared to 5, 4, and 3, as described in Figure 1b. However, the astaxanthin level decreased with the increase in nitrogen availability in the culture media, with maximum values recorded at 1 mM nitrate (8.4 mg astaxanthin/g dry biomass) and minimum values at 10 mM nitrate (0.2 mg astaxanthin/g dry biomass). Therefore, astaxanthin accumulation is maximized under limiting nutritional conditions or environmental stress. These findings align with the notion that nitrogen availability is a key factor for inducing astaxanthin accumulation in *C. zofingiensis*, consistent with previous considerations [40], and analogously to the situation in other astaxanthin-accumulating microalgae, such as *H. pluvialis* [20].

The relative cellular levels of astaxanthin and lutein in *C. zofingiensis* are related to the N content of the biomass. Different astaxanthin and lutein contents in cells were observed in cultures grown at a dilution rate of 0.5 d^−1^ with different N content in the biomass, reflecting the differences in nitrate concentration in the culture media (Figure 2b). It can be seen that the maximum astaxanthin content occurred when the cellular N content was minimal (about 4%), reaching values greater than 0.5 mg astaxanthin/g dry biomass, and decreasing progressively as the N content increased to attain a value of 0.03 mg/g at 9% N. The lutein content followed an inverse pattern to that of astaxanthin, increasing as the cellular N content rose, reaching values greater than 3 mg lutein/g dry biomass at a high N content.

Another way to analyze the relationship between nitrate supply and astaxanthin and lutein accumulation is by referring the latter to the specific nitrate input (SNI), as described in Materials and Methods. SNI influenced the productivity of the cultures in astaxanthin and lutein (g carotenoid per g dry biomass and per day) (Figure 2c). The highest astaxanthin productivity occurred when the SNI was at its lowest (1.2 mmol N/g biomass per day), reaching concentrations of 1.22 mg/g dry biomass per day. Conversely, when the SNI approached 3.8 mmol N/g biomass per day, astaxanthin productivity dropped to 0.07 mg/g dry biomass per day. In contrast, lutein productivity exhibited an opposite trend. At the same SNI, it increased until it reached 8.2 mg/g dry biomass per day.

These results are consistent with previous reports indicating a sharp increase in astaxanthin content in *C. zofingiensis* and a reduction in lutein content under conditions of low nitrogen availability [27,40] or a high C/N ratio [41].

### 2.3. High Irradiance Increases Astaxanthin Levels Only Under Nitrogen Limitation

Incident irradiance on the culture’s surface is not the same as that available to cells inside the reactor, for which the term average irradiance (Iav) is the most appropriate. The average irradiance determines the availability of light to the average cell in the culture. It is a function of the irradiance impinging at the reactor surface, the biomass concentration, the light path of the reactor, and the extinction coefficient of the biomass [42].

The effect of the average irradiance on lutein and astaxanthin content in *C. zofingiensis* was studied in continuous cultures at a dilution rate of 0.5 d^−1^ and two nitrate concentrations (1 and 10 mM) in the culture media.

**Figure 3:**
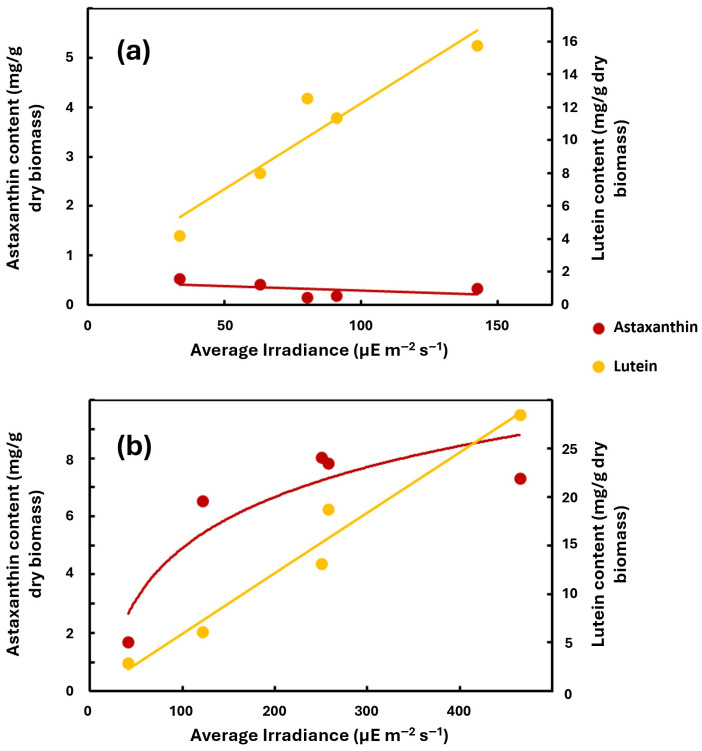
Effect of average irradiance on astaxanthin (red) and lutein (yellow) content in the biomass in continuous culture grown at 10 mM (panel (**a**)) and 1 mM (panel (**b**)) nitrate in the culture media.

For 10 mM NaNO_3_ in the culture media, the increase in average irradiance resulted in increased lutein content, reaching 15.7 mg lutein/g at a value of 142 µE m^−2^ s^−1^ (Figure 3a). On the contrary, the astaxanthin level remained below 1 g/g, being virtually unaffected by the increase in average irradiance.

As we have just seen, changes in available irradiance affect the cellular content of *C. zofingiensis* in lutein but not in astaxanthin when N availability is not limiting. Nevertheless, the situation changes as N availability is limited (1 mM NaNO_3_ in culture media), with the accumulation of both carotenoids, astaxanthin and lutein, being responsive to light availability (Figure 3b). At an Iav value close to 500 µE m^−2^ s^−1^, lutein in dry biomass reached a value close to 30 mg/g and astaxanthin increased over 7 mg/g. Therefore, we can conclude that astaxanthin accumulation only shows a dependence on irradiance when nitrogen is limiting for cells but not when this deprivation does not exist.

### 2.4. Synergistic Effect of Nitrate and Light Stress Modulates Astaxanthin and Lutein Content Through Differential Expression of Specific Genes

Transcriptome analysis can assist the elucidation of biological processes in a global way and is receiving increasing interest in algal research. Recently, we have unveiled the molecular mechanisms that control astaxanthin biosynthesis in *H. pluvialis* [20]. Applying this experience, we used RNA-seq to relate changes at the transcriptomic level in *C. zofingiensis* to changes in the accumulation of astaxanthin and lutein in this microalga. In addition, the complete genome sequence of *C. zofingiensis* is available [29] and its annotation has been improved recently [40], which facilitates robust and biologically meaningful analysis.

Comparing each condition using LL 10 mM as a control unstressed condition, large differences were detected in the number of differentially expressed genes. High irradiance (HL 10 mM) individually activated 76 genes and repressed 179. Nitrogen deprivation (LL 1 mM) increased these numbers to 693 activated and 447 repressed genes. Finally, the combination of both (HL 1 mM) led to the maximum impact, with 974 activated and 637 repressed genes. All conditions produced a repression of genes related to photosynthetic processes, while the activated genes were involved in different processes depending on the specific condition (Supplementary Figure S2). HL 10 mM-activated genes did not show significant enrichment in any biological process, while those in LL 1 mM were related to fatty acid biosynthesis, transport processes, and carbohydrate metabolism. The combined HL 1 mM condition showed activation in genes involved in processes similar to those in LL 1 mM, as well as mitotic and cytoskeletal organization processes.

**Figure 4:**
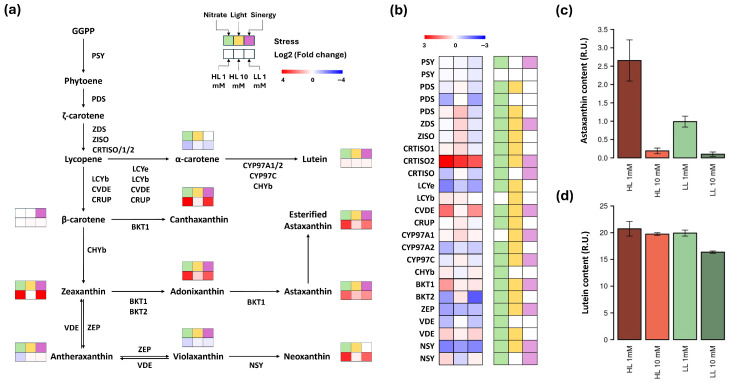
Transcriptomic and carotenoid profiling of carotenoid biosynthetic pathway. (**a**) Carotenoid biosynthetic pathway with the enzymes involved in each step. The content of each pigment is represented as the fold change in the comparison between each condition and the LL 10 mM control, with the order indicated in the legend. The significance of the effect of nitrate, light, and the synergy of the two are represented in green, yellow, and purple, respectively. (**b**) Expression level of each of the genes encoding for the enzymes in the pathway and the significance of their response to each stress, which has the same color code as in panel (**a**). (**c**) Astaxanthin content in each of the four conditions, measured in relative units of peak area. (**d**) Lutein content in each of the four conditions, measured in relative units of peak area.

In *C. zofingiensis*, all enzymes involved in the methylerythritol phosphate (MEP) pathway have been identified as encoded by single-copy genes [40]. Carotenoid synthesis in this microalga seems to depend on isoprenoids derived from this pathway rather than those from the mevalonate (MVA) one. It has been demonstrated that, under nitrogen deficiency, upregulation of MEP pathway does not occur [40], probably because it is not required for precursor supply, in contrast to the situation in *H. pluvialis* [20]. Our results are in agreement with these observations [41], since nitrogen limitation increases the astaxanthin content without affecting the total carotenoid content. A transcriptomic analysis of the genes encoding the enzymes involved in the carotenoid biosynthesis pathway and the contents of major carotenoids has been carried out, combining high (HL) and low (LL) light intensities with low (1 mM) and high (10 mM) nitrate concentrations in the culture media, using LL 10 mM as a control unstressed condition for all comparisons (Figure 4).

Under the low nitrate condition, an increase in the levels of secondary carotenoid content, like astaxanthin, canthaxanthin and adonixanthin, was apparent (Figure 4a), while, in the α-carotene branch, a strong reduction in α-carotene content and an increased accumulation of lutein occurred. In relation to light, the effects were similar but of a lower magnitude than those exerted by nitrate, so that high light intensities mimicked low nitrate concentrations to a reduced extent. Furthermore, a complementarity of both factors is patent, since their combination in the 1 mM HL samples gave rise to the most significant changes.

The channeling of metabolic flux to secondary carotenoids could be related to the levels of cyclases that transform lycopene into α- or β-carotene (Figure 4b). While β-cyclases (LCYb, CVDE and CRUP) were upregulated in all conditions with respect to the control, ε-cyclase LCYe, required for α-carotene production, was strongly repressed. Thus, α-carotene production was decreased, and the available lycopene was funneled to the β-carotene branch. However, the increased expression of the hydroxylase responsible for lutein production CYP97A1 under all stress conditions, as well as CYP97C under HL conditions, would explain the slight increases observed in lutein levels by altering the lutein/α-carotene ratio. Other studies have indicated that the reduced lutein level was related to the repression of CYP97A and CYP97C in certain stressful situations [34]. The present study would confirm that the upregulation of these enzymes could lead to lutein accumulation without compromising astaxanthin content (Figure 4c,d). Regarding astaxanthin accumulation, the lack of change in β-carotene levels is noteworthy. This behavior could be once again explained by the expression pattern of the CHYb hydroxylase responsible for zeaxanthin production. The gene encoding this enzyme increased its expression under low nitrate conditions while not undergoing significant changes when subjected to HL, with an increase in zeaxanthin levels and a reduction in the β-carotene content. When subjected to HL, a slight increase in β-carotene levels was observed, correlating with the absence of a change in CHYb expression and the maintenance of zeaxanthin levels. Although the immediate activation of CHYb in response to HL pulses is a widely supported phenomenon, its inhibition upon prolonged exposure has been reported in previous studies [29].

On the other hand, BKT ketolases convert β-carotene into canthaxanthin, zeaxanthin into adonixanthin and the latter into astaxanthin. Recent studies with mutants of this protein [43] suggested that BKT1 was the enzyme responsible for the catalysis of most of these steps, whereas BKT2 should only have a role in the transformation of zeaxanthin to astaxanthin. However, the expression patterns of both genes in our study conditions showed a high correlation of BKT1 levels with those of adonixanthin and astaxanthin (0.96 and 0.98, respectively), while BKT2 correlated negatively with them (−0.70 and −0.63), suffering a significant inhibition in low nitrate conditions. Under HL stress exclusively, BKT1 overexpression was lower than under nitrate stress, while BKT2 was slightly upregulated. This resulted in smaller increases in the levels of these ketocarotenoids relative to those under nitrate stress, suggesting that BKT1 was primarily responsible for catalyzing these reactions. The lower catalytic role of BKT2 could be related to situations where BKT1 was not expressed but basal levels of astaxanthin were needed. This is in line with a previous report of trace amounts of astaxanthin when only BKT2 is active [43].

The underexpression of ZEP in all conditions and the overexpression of VDE and NSY upon nitrate stress led to an accumulation of zeaxanthin and neoxanthin in this condition at the expense of antheraxanthin and violaxanthin.

Based on the above data, an analysis was carried out to determine which factor had a greater effect on the biosynthesis of each carotenoid (Figure 4a,b). Moreover, it was studied whether, in the presence of both factors, the effect was merely additive or whether there was a synergy that modified the regulation. A generalized effect of both nitrate and light and a synergistic interaction between them were observed. A clear example of such an interaction occurred in astaxanthin, where the increase in its levels in HL 1 mM was greater than the sum of the separated effects of nitrate (LL 1 mM) and light (HL 10 mM) (Figure 4c).

In terms of the significance of the effect on the content of all carotenoids, nitrate concentration triggered a more significant effect than that exerted by light (Table S1). For individual genes, the effect of nitrate was predominant on ketolases responsible for producing ketocarotenoids. Regarding hydroxylases and cyclases, both effects had an overall similar influence, with one or the other being more significant depending on the specific enzymes (Table S1). Finally, although the interaction between both factors did not modify the regulation of cyclases, except for CVDE, it did affect the enzymes in charge of producing the final compounds of the pathway, such as CYP97A1, CYP97C and BKT1, allowing the integration of stress signals to alter their final accumulated amounts. It should be noted that, in steps where catalysis was carried out by several enzymes, as in the hydroxylation of α-carotene to lutein, some enzymes responded to nitrate concentration (CHYb), others to light intensity (CYP97A1) and others to both (CYP97A2 and CYP97C), which also occurred in the case of β-cyclases.

Taken together, these results point to a greater impact of nitrate concentration on the generation of secondary carotenoids without ruling out the role of irradiance, both as an independent factor and as a modulator of the response to nitrate, as it has been shown to boost salinity stress response [44]. Moreover, an orchestration of the gene expression of the different enzymes is postulated to explain the final accumulation of the compounds of interest, lutein and astaxanthin, in the different conditions under study. Specifically, nitrate deprivation causes the channeling of carbon flux towards β-carotene through the control of the corresponding cyclases and its transformation into astaxanthin via the overexpression of BKT1. Additionally, high irradiance transforms the limited remnant of α-carotene into lutein by stimulating the expression of CYP97A1 and CYP97C. This results in the co-accumulation of lutein and astaxanthin, whose ratio can be adjusted based on nitrate content in the culture media and irradiance to achieve the desired values.

## 3. Materials and Methods

### 3.1. Strain and Culture Conditions

The microalga *C. zofingiensis* CCAP 211/14 was obtained from the Culture Collection of Algae and Protozoa, Ambleside (UK). Arnon medium [45] was modified to contain 4 mM K_2_HPO_4_ and different NaNO_3_ concentrations.

Experiments were performed in glass photobioreactors with 2 L capacity (0.07 m diameter, 0.5 m height) containing 1.8 L cell suspension. Agitation was provided by continuous bubbling air (~30 L per L of culture per h). The temperature of the culture was maintained at 25 °C by circulating water in reactor jacket. pH was maintained at 7.5 by pure CO_2_ injection on demand, using a pH-stat system. The cultures were illuminated by means of six white-light lamps (Osram ecopack-FQ24W/840HO, Munich, Germany) surrounding the photobioreactor. The irradiance impinging on the reactor surface was regulated by an automated system to simulate a solar neutral cycle (12 h light/12 h dark) increasing gradually from dark until a maximum irradiance (1000 µE m^−2^ s^−1^) on the reactor surface and decreasing again until dark. The incident irradiance (Io) was measured using a 4π quantum scalar irradiance sensor QSL-100 (Biospherical Instrument, San Diego, CA, USA). Six photobioreactors were inoculated simultaneously with batch-grown cells and operated on a batch regime for 3 days to reach an appropriate cell density. Henceforth, the reactors were switched to operate in continuous mode feeding continuously with culture media at different dilution rates (0.1; 0.3; 0.5; 0.7 and 0.9 d^−1^) only during the period of illumination. For each dilution rate, 6 different nitrate concentrations in the culture media (1; 2; 3; 4; 5 and 10 mM NO_3_Na) were tested. Samples for analytical determinations were collected 4 h after the beginning of the light period for at least four consecutive days of steady state and analyzed by duplicate.

Equation (1) was used to determine the average irradiance (Iav) as a measure of light availability for the cells, which is a function of the incident irradiance in the reactor (Io), biomass concentration (Cb), path of the light (p), and extinction coefficient (Ka) [42]. The value of the extinction coefficient for this microalga (as a function of the pigment content of the biomass) was 0.1. Nitrate availability was quantified as specific nitrate input (SNI) according to Del Rio et al. [46], where D is the dilution rate, as in Equation (2).

(1) Iav = Io/Ka·p·Cb (1 − exp ^(−Ka·pCb)^)

(2) SNI = [NO_3_^−^]·D/Cb

For transcriptomic analysis, *C. zofingiensis* was grown at a fixed dilution rate of 0.5 d^−1^ under two nitrate concentrations in the culture media (1 and 10 mM) and two maximum irradiance values on the reactor surface: 1000 µE m^−2^ s^−1^ for high light condition (HL) and 300 µE m^−2^ s^−1^ for low light condition (LL). Samples for analysis were collected for three consecutive days at 4 h after the start of the light cycle and analyzed by triplicate.

### 3.2. Analytical Methods

Biomass concentration was determined by dry weight measurement. For this purpose, 5–10 mL (per duplicate) aliquots of the cell suspension were filtered through a 1.2 µm Whatman GF/C filter paper, washed with distilled water, and the filters containing the cells were dried in an oven at 80 °C for 24 h. Astaxanthin, lutein and total carotenoid content of *C. zofingiensis* were determined in extracted cells of lyophilized biomass.

Carotenoid extraction was achieved following the method proposed by Del Campo et al. [27]. Darkness was maintained during the entire process to avoid pigment degradation. In total, 5 mg of lyophilized biomass was added to a hermetic tube containing 1 mL of glass beads (0.25 mm diameter) and 1 mL of pure acetone. Three disrupting cycles (60 s agitation–60 s incubation on ice) were applied using Mini-Beadbeater (BioSpec Products, Bartlesville, OK, USA). After centrifugation for 5 min at 13,000× *g*, cellular extracts were collected and saved in new tubes. Again, 1 mL of pure acetone was added to wash glass beads, which were then centrifuged, and the supernatant was collected. This process was repeated until the supernatant turned colorless. Supernatants were pooled together in the same tube and acetone was evaporated using a nitrogen gas stream. Finally, 350 μL of acetone was added for HPLC analysis, as previously described [27]. Values shown are mean ± SD of at least four determinations per duplicate.

Nitrogen present in the biomass was determined by elemental analysis of washed and dried samples using a CHNS-O elemental analyzer (FlashEA 1112 Series, Thermo, Rodano, Italy).

Nitrate concentration in the medium was quantified spectrophotometrically at 210 nm in the supernatant resulting from centrifugation of the cell suspension (3000× *g*, 5 min), as previously described [47].

### 3.3. RNA-Seq Data Generation and Processing

RNA extraction was performed using mechanical disruption of the frozen cell pellets in a Mini Bead Beater (Biospec Products) mixed with 0.5 mm glass beads in the presence of an extraction buffer consisting of phenol:chloroform (1:1, *v*/*v*). Subsequently, RNA was purified using ISOLATE II RNA Plant Kit (Bioline, London, UK) following manufacturer’s instructions. RNA integrity number (RIN) was computed using an Agilent 2100 Bioanalyzer (Agilent Technologies, Santa Clara, CA, USA) producing values greater than 8 per sample. Sequencing libraries were generated according to Illumina TruSeq Stranded mRNA protocol and sequenced on an Illumina NextSeq 500 sequencer (San Diego, CA, USA) producing approximately 17 million 150 nt long reads per sample.

Quantification of gene expression from raw reads was performed using the computational pipeline MARACAS [48], selecting *C. zofingiensis* genome sequence assembly and annotation v5.6 as reference genome [29]. Software used for quality control, read mapping, transcript assembly and gene expression quantification during the pipeline were FastQC v0.12.0, HISAT2 (v2.1) and StringTie (v.2.2.1), respectively [49]. Quantile normalization was determined to be the most adequate for our data, based on the results of Bioconductor R package NormalyzerDE(v1.28.0) [50]. The presence of low levels of noise in our three replicates was confirmed using Principal Component Analysis (PCA) (Figure S1). We obtained a high overall alignment rate, which suggests that sequencing had been performed accurately and that most reads had been correctly mapped to their corresponding genomic location. Differentially expressed genes were determined based on a fold change threshold and adjusted *p*-value thresholds of 2 and 0.05, respectively. Gene Ontology enrichment analysis was performed for BP GO terms based on the corresponding genome version annotation from Phytozome [51] and employing an adjusted *p*-value threshold of 0.05.

The effects of each factor and their interplay were determined using two-way ANOVA analysis with interaction on the levels of carotenoids and the genes involved in their biosynthesis. Genes identifiers were retrieved from the literature and Phytozome [51]. ANOVA assumptions of normality and homoscedasticity were tested using the Kolmogorov–Smirnoff test and the Levene test, respectively. For cases where the interaction was not significant, the test was recomputed without the interaction factor to better estimate the *p*-value associated with nitrate and light. The correlation between gene expression levels and carotenoid content was determined using Pearson’s method from the mean expression and mean carotenoid content matrices in each condition.

## Data Availability

RNA-seq data generated in this study will be freely available from the Gene Expression Omnibus (GEO) database under the accession number GSE317028. The data analysis code developed using the statistical programming language R is freely available from the following GitHub repository https://github.com/ramosgonzmarc/Cz (accessed on 10 March 2026).

## Supplementary Materials

The following supporting information can be downloaded at: https://www.mdpi.com/article/10.3390/plants15060902/s1, Table S1: Significance analysis for the effect of light, nitrate and their interaction on the levels of the different carotenoids and main carotenogenic enzymes. Both gene IDs and common names are provided for the latter. First three columns indicate the corresponding *p*-values from ANOVA analysis of each factor.; Figure S1: Principal Component Analysis showing global gene expression similarity of the different samples grouped by condition.; Figure S2: Tree plots showing enriched BP GO terms in comparison with LL 10 mM condition. (a) Repressed genes. From left to right: HL 10 mM, LL 1 mM and HL 1 mM. (b) Activated genes. From left to right: LL 1 mM, HL 1 mM.

## References

[1] Del Río E., Acién F.G., Guerrero M.G., Singh O.V., Harvey S.P. (2010). Photoautotrophic Production of Astaxanthin by the Microalga *Haematococcus pluvialis*. Sustainable Biotechnology: Sources of Renewable Energy.

[2] Chisti Y. (2007). Biodiesel from Microalgae. Biotechnol. Adv..

[3] Wijffels R.H., Barbosa M.J. (2010). An Outlook on Microalgal Biofuels. Science.

[4] Hu Q., Sommerfeld M., Jarvis E., Ghirardi M., Posewitz M., Seibert M., Darzins A. (2008). Microalgal Triacylglycerols as Feedstocks for Biofuel Production: Perspectives and Advances. Plant J..

[5] Demmig-Adams B., Adams W.W. (2002). Antioxidants in Photosynthesis and Human Nutrition. Science.

[6] Sun T., Rao S., Zhou X., Li L. (2022). Plant Carotenoids: Recent Advances and Future Perspectives. Mol. Hortic..

[7] Gómez-Sagasti M.T., López-Pozo M., Artetxe U., Becerril J.M., Hernández A., García-Plazaola J.I., Esteban R. (2023). Carotenoids and Their Derivatives: A “Swiss Army Knife-like” Multifunctional Tool for Fine-Tuning Plant-Environment Interactions. Environ. Exp. Bot..

[8] Dose J., Matsugo S., Yokokawa H., Koshida Y., Okazaki S., Seidel U., Eggersdorfer M., Rimbach G., Esatbeyoglu T. (2016). Free Radical Scavenging and Cellular Antioxidant Properties of Astaxanthin. Int. J. Mol. Sci..

[9] Ambati R.R., Moi P.S., Ravi S., Aswathanarayana R.G. (2014). Astaxanthin: Sources, Extraction, Stability, Biological Activities and Its Commercial Applications—A Review. Mar. Drugs.

[10] Lorenz R.T., Cysewski G.R. (2000). Commercial Potential for Haematococcus Microalgae as a Natural Source of Astaxanthin. Trends Biotechnol..

[11] Donoso A., González-Durán J., Muñoz A.A., González P.A., Agurto-Muñoz C. (2021). Therapeutic Uses of Natural Astaxanthin: An Evidence-Based Review Focused on Human Clinical Trials. Pharmacol. Res..

[12] Fumia A., Cicero N., Gitto M., Nicosia N., Alesci A. (2021). Role of Nutraceuticals on Neurodegenerative Diseases: Neuroprotective and Immunomodulant Activity. Nat. Prod. Res..

[13] Rao A.V., Rao L.G. (2007). Carotenoids and Human Health. Pharmacol. Res..

[14] Li L.H., Lee J.C.Y., Leung H.H., Lam W.C., Fu Z., Lo A.C.Y. (2020). Lutein Supplementation for Eye Diseases. Nutrients.

[15] Feng L., Nie K., Jiang H., Fan W. (2019). Effects of Lutein Supplementation in Agerelated Macular Degeneration. PLoS ONE.

[16] van Bergeijk S.A., Paullada Salmerón J.A., López Pérez A.I., Moreno J., Cañavate J.P., García-González M. (2013). Lutein Enrichment of the Rotifer *Brachionus* sp. Using Freeze-Dried *Muriellopsis* sp. Cells. Aquac. Res..

[17] Lin J.-H., Lee D.-J., Chang J.-S. (2015). Lutein Production from Biomass: Marigold Flowers versus Microalgae. Bioresour. Technol..

[18] Fu Y., Wang Y., Yi L., Liu J., Yang S., Liu B., Chen F., Sun H. (2023). Lutein Production from Microalgae: A Review. Bioresour. Technol..

[19] Mota G.C.P., Moraes L.B.S.d., Oliveira C.Y.B., Oliveira D.W.S., Abreu J.L.d., Dantas D.M.M., Gálvez A.O. (2022). Astaxanthin from *Haematococcus pluvialis*: Processes, Applications, and Market. Prep. Biochem. Biotechnol..

[20] Hoys C., Romero-Losada A.B., del Río E., Guerrero M.G., Romero-Campero F.J., García-González M. (2021). Unveiling the Underlying Molecular Basis of Astaxanthin Accumulation in *Haematococcus* through Integrative Metabolomic-Transcriptomic Analysis. Bioresour. Technol..

[21] Raposo M.F.d.J., De Morais A.M.M.B., De Morais R.M.S.C. (2015). Carotenoids from Marine Microalgae: A Valuable Natural Source for the Prevention of Chronic Diseases. Mar. Drugs.

[22] Ip P.F., Wong K.H., Chen F. (2004). Enhanced Production of Astaxanthin by the Green Microalga *Chlorella zofingiensis* in Mixotrophic Culture. Process Biochem..

[23] Chen J.H., Wei D., Lim P.E. (2020). Enhanced Coproduction of Astaxanthin and Lipids by the Green Microalga *Chromochloris zofingiensis*: Selected Phytohormones as Positive Stimulators. Bioresour. Technol..

[24] Mulders K.J.M., Janssen J.H., Martens D.E., Wijffels R.H., Lamers P.P. (2014). Effect of Biomass Concentration on Secondary Carotenoids and Triacylglycerol (TAG) Accumulation in Nitrogen-Depleted *Chlorella zofingiensis*. Algal Res..

[25] Liu J., Sun Z., Zhong Y., Gerken H., Huang J., Chen F. (2013). Utilization of Cane Molasses towards Cost-Saving Astaxanthin Production by a *Chlorella zofingiensis* Mutant. J. Appl. Phycol..

[26] Sun N., Wang Y., Li Y.T., Huang J.C., Chen F. (2008). Sugar-Based Growth, Astaxanthin Accumulation and Carotenogenic Transcription of Heterotrophic *Chlorella zofingiensis* (Chlorophyta). Process Biochem..

[27] Del Campo J.A., Rodríguez H., Moreno J., Vargas M.Á., Rivas J., Guerrero M.G. (2004). Accumulation of Astaxanthin and Lutein in *Chlorella zofingiensis* (Chlorophyta). Appl. Microbiol. Biotechnol..

[28] Liu J., Sun Z., Gerken H., Liu Z., Jiang Y., Chen F. (2014). *Chlorella zofingiensis* as an Alternative Microalgal Producer of Astaxanthin: Biology and Industrial Potential. Mar. Drugs.

[29] Roth M.S., Cokus S.J., Gallaher S.D., Walter A., Lopez D., Erickson E., Endelman B., Westcott D., Larabell C.A., Merchant S.S. (2017). Chromosome-Level Genome Assembly and Transcriptome of the Green Alga *Chromochloris zofingiensis* Illuminates Astaxanthin Production. Proc. Natl. Acad. Sci. USA.

[30] Roth M.S., Gallaher S.D., Westcott D.J., Iwai M., Louie K.B., Mueller M., Walter A., Foflonker F., Bowen B.P., Ataii N.N. (2019). Regulation of Oxygenic Photosynthesis during Trophic Transitions in the Green Alga *Chromochloris zofingiensis*. Plant Cell.

[31] Huang W., Ye J., Zhang J., Lin Y., He M., Huang J. (2016). Transcriptome Analysis of *Chlorella zofingiensis* to Identify Genes and Their Expressions Involved in Astaxanthin and Triacylglycerol Biosynthesis. Algal Res..

[32] Ip P.-F., Chen F. (2005). Production of Astaxanthin by the Green Microalga *Chlorella zofingiensis* in the Dark. Process Biochem..

[33] Liu J., Mao X., Zhou W., Guarnieri M.T. (2016). Simultaneous Production of Triacylglycerol and High-Value Carotenoids by the Astaxanthin-Producing Oleaginous Green Microalga *Chlorella zofingiensis*. Bioresour. Technol..

[34] Zhang Y., Ye Y., Bai F., Liu J. (2021). The Oleaginous Astaxanthin-Producing Alga *Chromochloris zofingiensis*: Potential from Production to an Emerging Model for Studying Lipid Metabolism and Carotenogenesis. BioMed Cent..

[35] Ali H.E.A., Vorisek F., Dowd S.E., Kesner S., Song Y., Qian D., Crocker M. (2022). Formation of Lutein, β-Carotene and Astaxanthin in a Coelastrella Sp. Isolate. Molecules.

[36] Shah M.d.M.R., Liang Y., Cheng J.J., Daroch M. (2016). Astaxanthin-Producing Green Microalga *Haematococcus pluvialis*: From Single Cell to High Value Commercial Products. Front. Plant Sci..

[37] Del Campo J.A., Moreno J., Rodríguez H., Angeles Vargas M., Rivas J., Guerrero M.G. (2000). Carotenoid Content of Chlorophycean Microalgae: Factors Determining Lutein Accumulation in *Muriellopsis* sp. (Chlorophyta). J. Biotechnol..

[38] Ambati R.R., Ravi S., Aswathanarayana R.G. (2010). Enhancement of Carotenoids in Green Alga-*Botryococcus braunii* in Various Autotrophic Media under Stress Conditions. Int. J. Biomed. Pharm. Sci..

[39] Minhas A.K., Hodgson P., Barrow C.J., Adholeya A. (2016). A Review on the Assessment of Stress Conditions for Simultaneous Production of Microalgal Lipids and Carotenoids. Front. Microbiol..

[40] Zhang Y., Shi M., Mao X., Kou Y., Liu J. (2019). Time-Resolved Carotenoid Profiling and Transcriptomic Analysis Reveal Mechanism of Carotenogenesis for Astaxanthin Synthesis in the Oleaginous Green Alga *Chromochloris zofingiensis*. Biotechnol. Biofuels.

[41] Chen Q., Chen Y., Hu Q., Han D. (2023). Metabolomic Analysis Reveals Astaxanthin Biosynthesis in Heterotrophic Microalga *Chromochloris zofingiensis*. Bioresour. Technol..

[42] Molina Grima E., Sánchez Pérez J.A., Garcia Camacho F., Garcia Sánchez J.L., López Alonso D. (1993). N-3 PUFA Productivity in Chemostat Cultures of Microalgae. Appl. Microbiol. Biotechnol..

[43] Ye Y., Huang J.-C. (2020). Defining the Biosynthesis of Ketocarotenoids in *Chromochloris zofingiensis*. Plant Divers..

[44] Kou Y., Liu M., Sun P., Dong Z., Liu J. (2020). High Light Boosts Salinity Stress-Induced Biosynthesis of Astaxanthin and Lipids in the Green Alga *Chromochloris zofingiensis*. Algal Res..

[45] Arnon D.I., McSwain B.D., Tsujimoto H.Y., Wada K. (1974). Photochemical Activity and Components of Membrane Preparations from Blue-Green Algae. I. Coexistence of Two Photosystems in Relation to Chlorophyll a and Removal of Phycocyanin. Biochim. Biophys. Acta-Bioenerg..

[46] Del Río E., Acién F.G., García-Malea M.C., Rivas J., Molina-Grima E., Guerrero M.G. (2005). Efficient One-Step Production of Astaxanthin by the Microalga *Haematococcus pluvialis* in Continuous Culture. Biotechnol. Bioeng..

[47] Cawse P.A. (1967). The Determination of Nitrate in Soil Solutions by Ultraviolet Spectrophotometry. Analyst.

[48] Romero-Losada A.B., Arvanitidou C., de los Reyes P., García-González M., Romero-Campero F.J. (2022). ALGAEFUN with MARACAS, MicroALGAE FUNctional Enrichment Tool for MicroAlgae RnA-Seq and Chip-Seq AnalysiS. BMC Bioinform..

[49] Pertea M., Kim D., Pertea G.M., Leek J.T., Salzberg S.L. (2016). Transcript-Level Expression Analysis of RNA-Seq Experiments with HISAT, StringTie and Ballgown. Nat. Protoc..

[50] Willforss J., Chawade A., Levander F. (2019). NormalyzerDE: Online Tool for Improved Normalization of Omics Expression Data and High-Sensitivity Differential Expression Analysis. J. Proteome Res..

[51] Goodstein D.M., Shu S., Howson R., Neupane R., Hayes R.D., Fazo J., Mitros T., Dirks W., Hellsten U., Putnam N. (2012). Phytozome: A Comparative Platform for Green Plant Genomics. Nucleic Acids Res..

