# Supplementary material for: Acute Combination of Nitrogen Deprivation and High Irradiance Induces the Simultaneous Accumulation of Astaxanthin and Lutein in Continuous Cultures of the Microalga Chromochloris zofingiensis
Source: Plants (Basel). 2026 Mar 14;15(6):902. doi: 10.3390/plants15060902 (PMC13029703; doi:10.3390/plants15060902)
Supplement: Supplementary file 1 [file plants-15-00902-s001.zip › Sup_Table_S1.pdf]

Table S1. Significance analysis for the effect of light, nitrate and their interaction on the levels of the different carotenoids and main carotenogenic enzymes. Both gene IDs and common names are provided for the latter. First three columns indicate the corresponding p-values from ANOVA analysis of each factor.

nitrate	light	interaction	assumptions	name	nit_sig	light_sig	int_sig	ids
**1.87E-03**	3.45E-05	0.42588569	TRUE	neoxanthin	*	*	-	neoxanthin
**3.29E-08**	2.65E-08	1.45E-06	FALSE	violaxanthin	*	*	*	violaxanthin
**8.62E-06**	6.52E-09	0.00017164	TRUE	astaxanthin	*	*	*	astaxanthin
**5.71E-08**	0.00012639	0.00505523	FALSE	lutein	*	*	*	lutein
**5.80E-00**	5.93E-03	5.93E-03	TRUE	zeaxanthin	*	*	*	zeaxanthin
**1.14E-01**	1.21E-05	1.42E-04	TRUE	canthaxantin	*	*	*	canthaxantin
**1.01E-01**	8.84E-03	1.28E-04	TRUE	astaxanthin_esterified	*	*	*	astaxanthin_esterified
**3.68E-02**	6.69E-06	2.50E-07	TRUE	adonixanthin	*	*	*	adonixanthin
**2.90E-08**	0.00113309	0.74004157	TRUE	a-carotene	*	*	-	a-carotene
**0.121232**	0.6232917	9.70E-09	FALSE	b-carotene	-	-	*	b-carotene
**3.83E-07**	0.0001653	0.00604979	TRUE	antheraxantion	*	*	*	antheraxantion
**0.00021797**	0.00060651	0.00933642	TRUE	BKT1	*	*	*	Cz13g13100
**3.17E-09**	0.04842294	0.5825	TRUE	BKT2	*	*	-	Cz04g11250
**0.06513144**	0.0009623	0.00050217	TRUE	CYP97A1	-	*	*	Cz13g16110
**3.86E-05**	3.62E-07	0.0833426	TRUE	CYP97A2	*	*	-	Cz09g14130
**3.43E-07**	0.00192263	0.00200967	TRUE	CYP97C	*	*	*	Cz09g07100
**0.03460289**	0.53291318	0.58009615	TRUE	CHYb	*	-	-	Cz12g16080
**0.04824649**	0.18328346	0.01142329	TRUE	PSY	*	-	*	Cz05g32220
**0.10778513**	0.36882949	0.1132702	TRUE	PSY	-	-	-	Cz05g32230
**5.73E-08**	0.0057856	0.58773753	TRUE	PDS	*	*	-	Cz02g32280
**3.13E-05**	0.12358769	0.42949902	TRUE	PDS	*	-	-	Cz07g17250
**0.00171484**	0.00026179	0.3900462	TRUE	PDS	*	*	-	Cz08g09110
**3.87E-08**	9.24E-08	0.00371019	TRUE	ZDS	*	*	*	Cz10g17010
**4.29E-09**	0.00024867	0.11350975	TRUE	ZISO	*	*	-	Cz10g17130
**3.95E-06**	0.17021665	0.00739225	TRUE	CRTISO	*	-	*	Cz16g01210
**0.00036343**	3.24E-08	0.55178057	TRUE	CRTISO1	*	*	-	Cz12g03260
**9.45E-07**	1.46E-07	0.03828593	TRUE	CRTISO2	*	*	*	Cz14g22040
**7.51E-06**	1.70E-09	0.0595653	TRUE	LCYe	*	*	-	Cz09g18310
**0.20661904**	9.97E-09	0.36201076	TRUE	LCYb	-	*	-	Cz12g10170
**0.00450304**	0.0020518	0.32084781	TRUE	CRUP	*	*	-	Cz07g07080
**1.00E-05**	0.00035122	0.01952858	TRUE	CVDE	*	*	*	Cz13g15030
**1.37E-08**	4.99E-08	0.00085119	TRUE	ZEP	*	*	*	Cz07g30060
**1.02E-06**	0.05693116	0.36738701	TRUE	VDE	*	-	-	Cz06g02070
**6.50E-07**	0.03220148	0.19367468	TRUE	VDE	*	*	-	Cz06g01120
**1.78E-09**	0.18901712	0.00025783	TRUE	NSY	*	-	*	Cz15g04070
**4.64E-08**	0.00041221	0.00075719	TRUE	NSY	*	*	*	Cz01g02290
