# Supplementary figures and images for: Acute Combination of Nitrogen Deprivation and High Irradiance Induces the Simultaneous Accumulation of Astaxanthin and Lutein in Continuous Cultures of the Microalga Chromochloris zofingiensis
Source: Plants (Basel). 2026 Mar 14;15(6):902. doi: 10.3390/plants15060902 (PMC13029703; doi:10.3390/plants15060902)

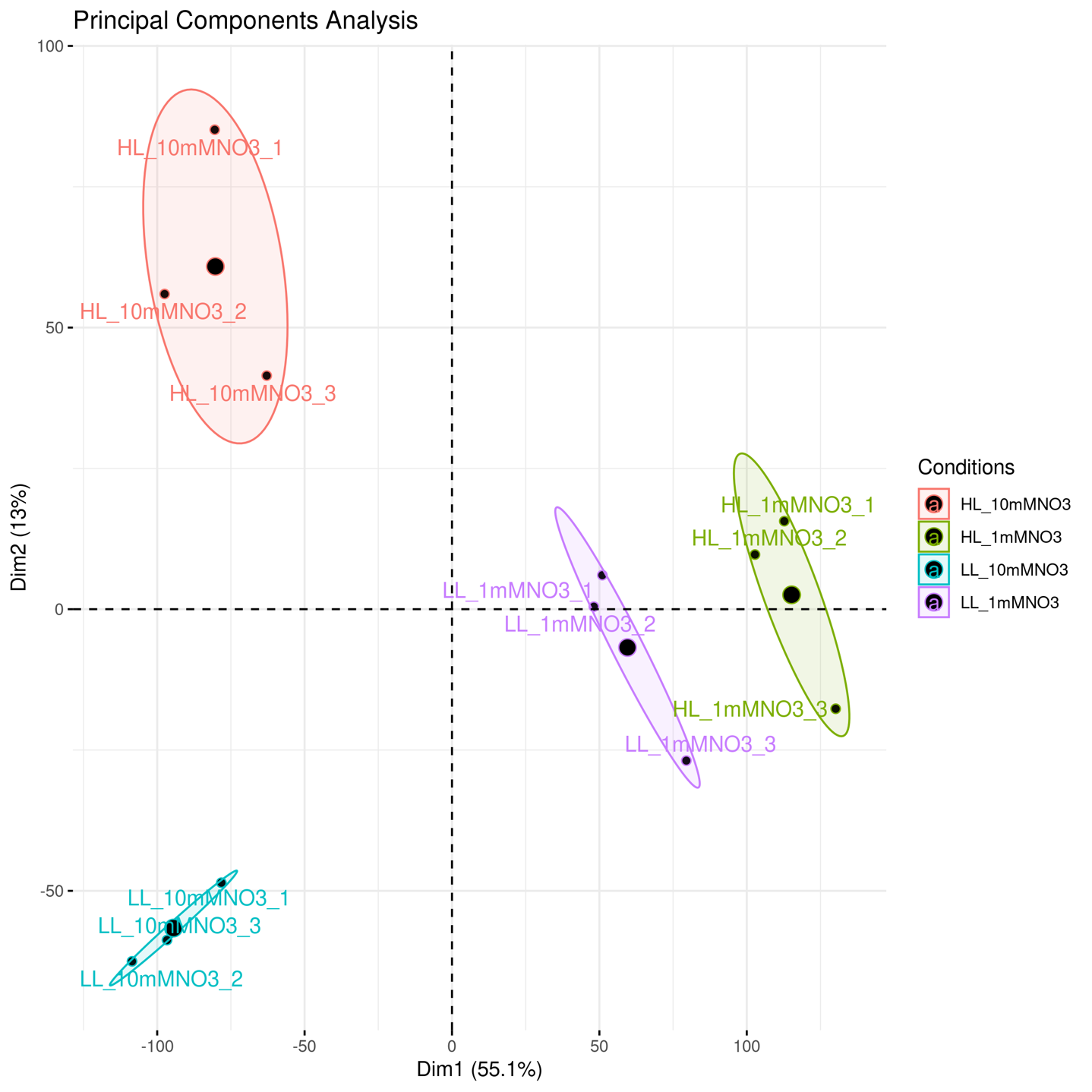

Supplement: Supplementary file 1 [file plants-15-00902-s001.zip › Sup_Figure_S1.tif]

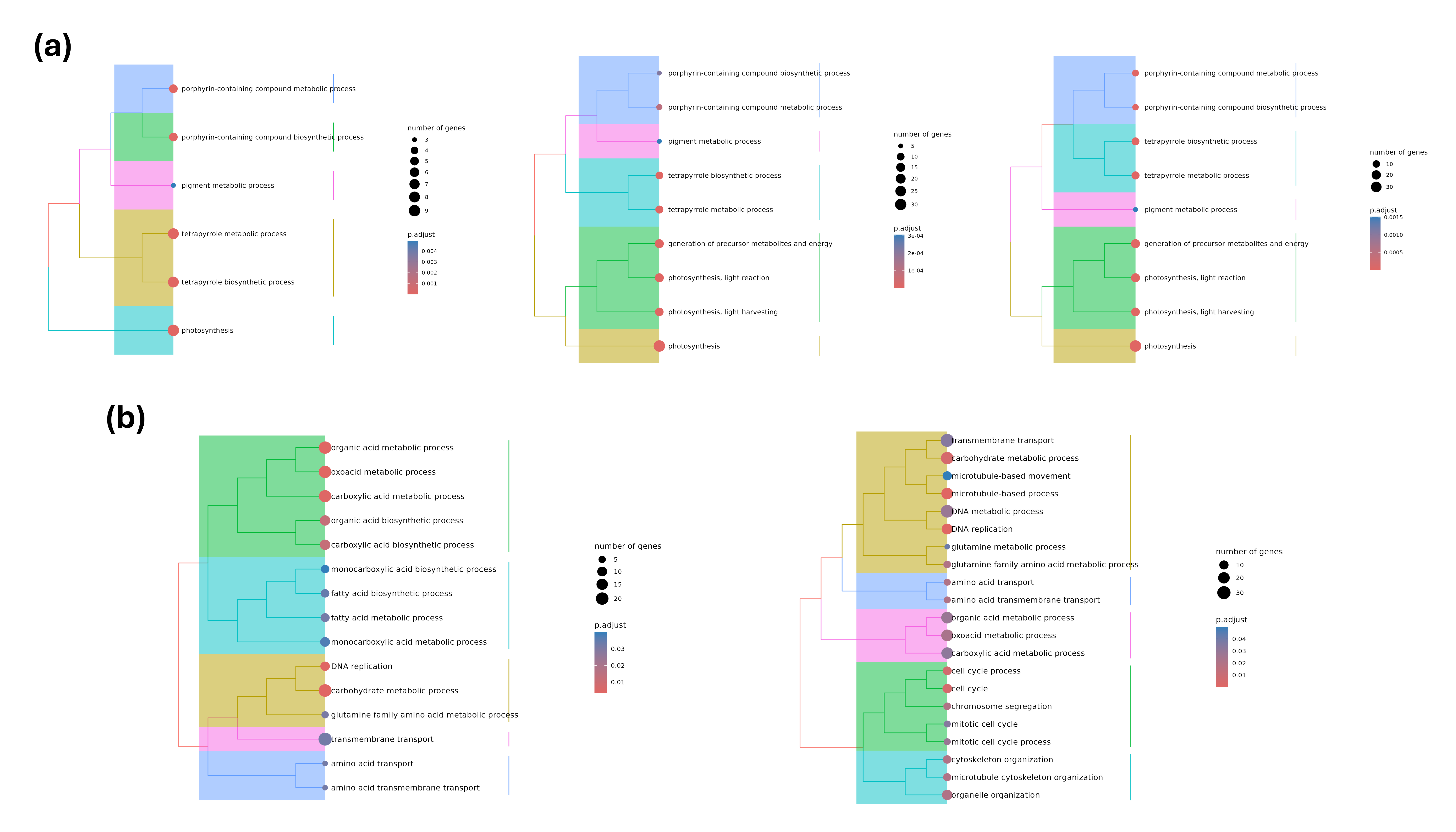

Supplement: Supplementary file 1 [file plants-15-00902-s001.zip › Sup_Figure_S2.tif]
